# Computer-aided diagnosis of external and middle ear conditions: A machine learning approach

**DOI:** 10.1371/journal.pone.0229226

**Published:** 2020-03-12

**Authors:** Michelle Viscaino, Juan C. Maass, Paul H. Delano, Mariela Torrente, Carlos Stott, Fernando Auat Cheein

**Affiliations:** 1 Department of Electronic Engineering, Universidad Técnica Federico Santa María, Valparaíso, Chile; 2 Interdisciplinary Program of Phisiology and Biophisics, Facultad de Medicina, Instituto de Ciencias Biomedicas, Universidad de Chile, Santiago, Chile; 3 Department of Neuroscience, Facultad de Medicina, Universidad de Chile, Santiago, Chile; 4 Department of Otolaryngology, Hospital Clínico de la Universidad de Chile, Santiago, Chile; Universidad de Salamanca, SPAIN

## Abstract

In medicine, a misdiagnosis or the absence of specialists can affect the patient’s health, leading to unnecessary tests and increasing the costs of healthcare. In particular, the lack of specialists in otolaryngology in third world countries forces patients to seek medical attention from general practitioners, whom might not have enough training and experience for making correct diagnosis in this field. To tackle this problem, we propose and test a computer-aided system based on machine learning models and image processing techniques for otoscopic examination, as a support for a more accurate diagnosis of ear conditions at primary care before specialist referral; in particular, for myringosclerosis, earwax plug, and chronic otitis media. To characterize the tympanic membrane and ear canal for each condition, we implemented three different feature extraction methods: color coherence vector, discrete cosine transform, and filter bank. We also considered three machine learning algorithms: support vector machine (SVM), k-nearest neighbor (k-NN) and decision trees to develop the ear condition predictor model. To conduct the research, our database included 160 images as testing set and 720 images as training and validation sets of 180 patients. We repeatedly trained the learning models using the training dataset and evaluated them using the validation dataset to thus obtain the best feature extraction method and learning model that produce the highest validation accuracy. The results showed that the SVM and k-NN presented the best performance followed by decision trees model. Finally, we performed a classification stage –i.e., diagnosis– using testing data, where the SVM model achieved an average classification accuracy of 93.9%, average sensitivity of 87.8%, average specificity of 95.9%, and average positive predictive value of 87.7%. The results show that this system might be used for general practitioners as a reference to make better decisions in the ear pathologies diagnosis.

## Introduction

In the last decade, the potential use of artificial intelligence (AI) in the field of medicine has grown exponentially. As an example, the first AI-based diagnostic device, called IDx-DR, was approved by the FDA to automatically detect retinopathy in patients with diabetes without requiring a clinician [1]. Medical imaging techniques such as computed tomography, magnetic resonance imaging, positron emission tomography, ultrasound and X-ray images, have facilitated the creation of large digital databases that can be processed with AI tools [2]. Thus, AI is now playing an important role in medical imaging interpretation to support tasks such as early detection, accurate diagnosis and treatment for diseases [3–5]. In addition, AI has powerful algorithms that can be used to enhance medical tasks and skills, thus overcoming fatigue, distraction, out of date in new diagnosis techniques or age-related impairment of the visual sense in physicians [6]. The most relevant applications of AI involve machine learning (ML) techniques which include enhancing cancer diagnosis [7]: the human physician error rate was reduced by 85% in metastatic breast cancer detection [8]; improve early detection of polyps to prevent colorectal cancer [9, 10], with accuracy achieved greater than 98%; and classification of tissues and subsequent recognition of cardiovascular organs [11]. Other promising applications include the rapid identification of radiographic anomalies [12], delineation of surgical anatomy [13] and classification of malignant tissues in pathologic specimens [14], assisted by computer vision techniques.

There are still open challenges in a wide diversity of medical applications, such is the case of otolaryngology [15]. In this context, some efforts focused on head and neck oncology and the use of ML to classify malignant tissue based on radiographic and histopathologic features [16–18]. Other studies have been developed for inner ear disorders [19, 20], classification of hearing loss phenotypes [21] and vocal fold diagnoses [22]. However, the use of AI tools for external or middle ear diseases is still at early stages of research. Some studies [23, 24] have developed computer-aided systems based on a binary classification approach. They used color information of the eardrum image to train different learning models –e.g., decision trees, SVM, neural networks and Bayesian decision approaches– and to predict if the image corresponds to a normal ear or otitis media case. The same pathologies were also addressed in [25], the authors included color, texture and shape information using three local descriptors: grid color moment, local binary pattern and histograms of oriented gradient. Furthermore, the classification stage was made using classical SVM approach. A more complete study to classify middle ear diseases was presented in [26], which included specific information for diseases such as tympanosclerosis, perforation, cerumen, retraction and post-injection crust. Other studies such as [27] and [28] proposed smartphone-based diagnostic system. In particular, [27] used an active contour for segmentation of region of interest (ROI) in the image, and then computer vision algorithms were applied to discriminate between different otitis media cases. The system was implemented in a server that received the images from the device. The other work, [28], used a neural network to classify the normal and otitis media cases. The images were acquired via a portable digital otoscope, then they were stored and visualized on a smartphone as well, but the images were previously processed on a dedicated server. Another related study [29] proposed a deep learning model for detection and segmentation of the structures of the tympanic membrane. Although the aim of such study is not conducted to support or help diagnose, the positive rate detection of structures of the tympanic membrane was the highest reported up to date: 93.1%. Recently, convolutional neural networks (CNNs) were used to otitis media diagnosis tasks [30, 31]. The authors in [30] employed a transfer learning approach to classify between four classes: normal, acute otitis media, chronic suppurative otitis media, and earwax. The two main drawbacks of this study were ensuring sufficient data to generate a reproducible model and model bias due to the image acquisition process –only one device was used to acquire the images. The authors in [31] developed a CNN model to binary classification between normal and otitis media cases, and also to analyze the presence or absence of perforation on the tympanic membrane achieving an accuracy of 91%.

External and middle ear diseases are among the most frequent pathologies treated by general practitioners, especially in the childhood [32]. Currently, the diagnosis of these diseases is carried out by the medical interview and a physical examination of the ear assisted by a manual otoscope. Otolaryngologists also use other instruments such as oto-endoscopes or oto-microscopes to improve the diagnosis. Such instruments are expensive, and therefore clinics have a limited number of them. Accordingly, only the most complex cases of ear diseases are examined using such instruments. In third world countries, the situation gets worse due to the lack of specialists, and general practitioners face the challenge of making an accurate and reliable diagnosis. To improve the diagnosis accuracy and to reduce the subjectivity involved in the general practitioner skills, we propose and test a computer-aided system based on machine learning models and image processing techniques as a support for fast diagnosis of ear conditions at primary care before specialist referral. The proposed system involves the use of a digital otoscope which has been introduced to the general practitioners’ daily activities. Since digital otoscope is a computer-aided vision system, images and video streaming are available for further processing. We consider a multi-class image classification to distinguish among earwax plug, myringosclerosis, chronic otitis media and identify normal cases. To generate the feature space, we use color and texture information for the pixel-level characterization of an RGB (red-green-blue) image. Then, several trained models based on machine learning techniques are used to classify the image. Finally, the output of the classification stage is a label that can be interpreted as a diagnosis delivered by the computer-aided system.

## Materials and methods

**Fig 1:**
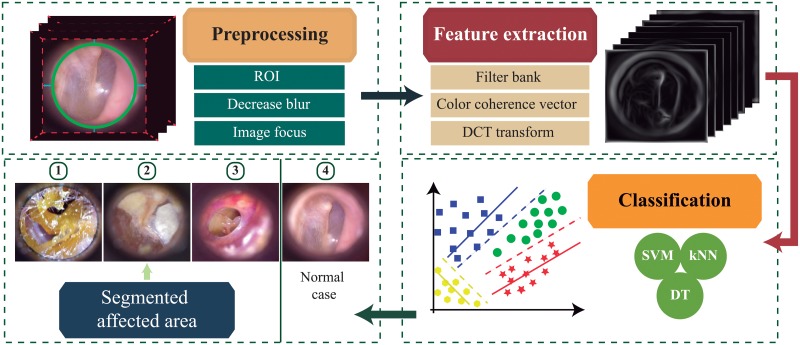
General scheme of the computer-aided system approach to assist the diagnosis of external and middle ear conditions. We selected the feature extraction method and classifier that produce the highest validation accuracy to use in our proposed system. The area under medical suspicion is highlighted for visualization purposes for general practitioner.

The general architecture of the computer-aided diagnosis system implemented in this work is summarized in Fig 1. The system receives a single RGB image and makes a prediction among the following ear classes: normal, myringosclerosis, earwax plug and chronic otitis media. If the prediction corresponds to an ear condition, the system highlights the specific area under medical suspicion on the image. Otherwise, it labels the image as a normal ear. To make the predictions, we train several classification models using the extracted features of the RGB image. Following, each stage from Fig 1 is explained in detail.

### Ear imagery database

The database employed in our experiments was created in collaboration with the Department of Otolaryngology of the Clinical Hospital from *Universidad de Chile*. To conduct the procedures, the study protocol number 65 (996/18) was reviewed and approved by the Scientific or Research Ethics Committee of the Clinical Hospital from *Universidad de Chile*. All patients and caregivers –under 18 years– gave written informed consent and all procedures were conducted in accordance with this protocol, to national regulations and the declaration of Helsinki, revision 2013.

#### Participants

A total of 180 subjects ranging from 7 to 65 years old were recruited after spontaneous consultation to the otolaryngology outpatient clinic of the Clinical Hospital from *Universidad de Chile*. All the participants were evaluated by an otolaryngologist, signed a written informed consent to participate, and met just one of the four following conditions at otoscopy: earwax plug, myringosclerosis, chronic otitis media or a normal otoscopy.

**Fig 2:**
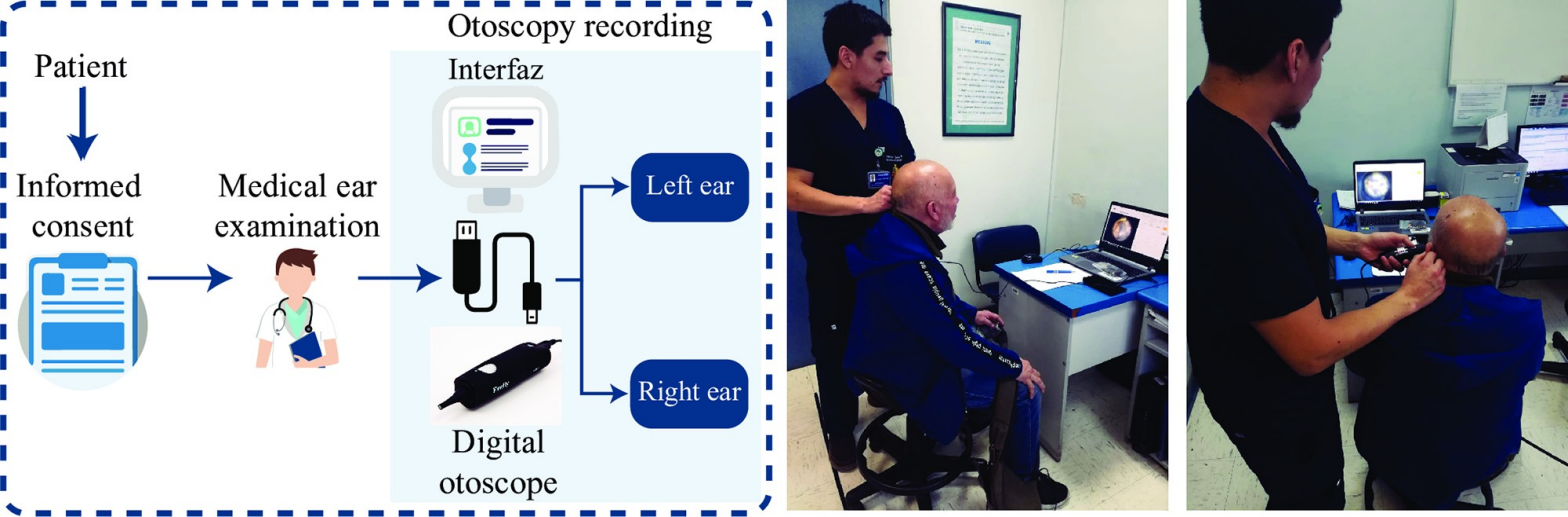
General procedure to obtain video otoscopies: The patient after signing the informed consent goes to one of boxes of the Clinical Hospital from *Universidad de Chile*. The physician performs the examination using a digital otoscope and the records are saved in a computer. The right and left ear examination using the system developed is shown.

Ear examinations were carried out using a digital otoscope DE500 Firefly –with a 0.4 mm and 0.5 mm speculums– connected to a computer. We acquired the video files at 20 FPS (frames per second) with a 640×480 pixels resolution. The physician was capable of visualizing the video acquired by the digital otoscope in real-time, on the computer screen. For this purpose and with the aim of recording all cases, a user interface was developed for the specialist, to minimize operational delays associated with the use of the equipment. Such an interface also allowed to record the physician’s diagnosis. The general procedure followed is shown in Fig 2—the individual in this figure has given written informed consent (as outlined in PLOS consent form) to publish these procedure details.

### Data pre-processing

Based on the physician’s diagnosis, we selected 90 video otoscopies for each condition –earwax plug, myringoesclerosis and chronic otitis media– and the same number of videos for normal cases.

Owing to we considered RGB still images of the eardrum as the input of the system, we extracted the frames of each recording and selected the best frames. This selection was carried out according to a blurriness threshold and was validated by the specialist. The blurriness in images was evaluated using the variance of the Laplacian method, which consists of taking a single channel of an image and convolving it with the Laplacian Kernel. Then it takes the variance of the response; if the variance falls below a pre-defined threshold, then the image is considered blurry and therefore, discarded. The Laplacian operator highlights regions of an image containing rapid intensity changes (edges). Therefore, the method assumes that if an image contains high variance then the responses correspond to an in-focused image, whereas if the variance is low, then the image is blurred. More information about this technique can be found in [33].

**Fig 3:**
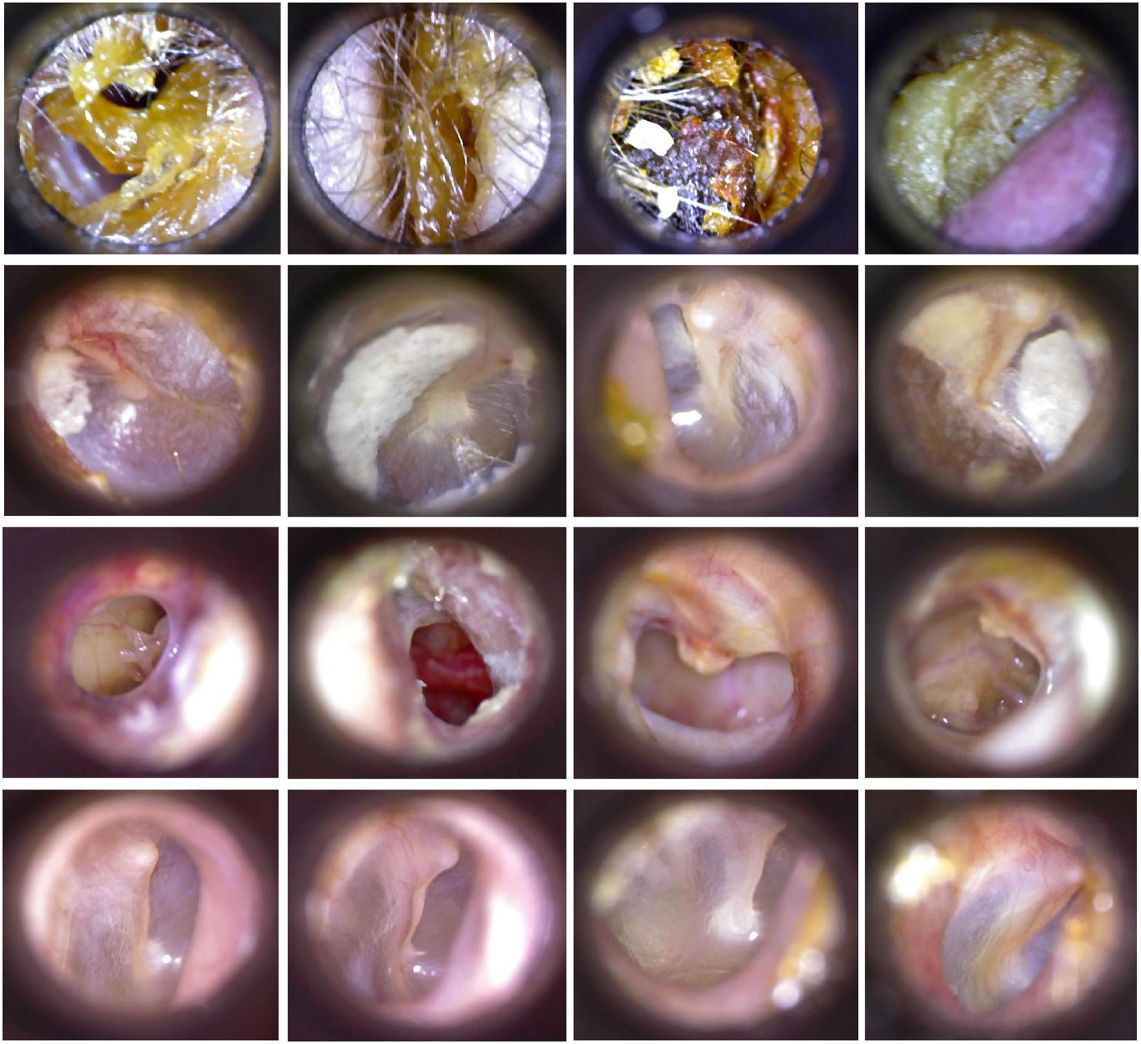
Ear imagery database per class. By rows, from top to bottom: earwax cases, myringoesclerosis cases, chronic otitis media cases and normal cases.

After selecting the best frames from each video, we isolated the ROI that included the eardrum. The ROI can be in any location in the whole image due to the physical characteristics of the otoscope. Accordingly, we first found the circle –using a circular Hough transform, shown in [34]– that represents the tip of the otoscope and then cropped the image using a pre-defined size. Finally, we obtained an image of 420×380 pixels where the ROI was centered. Some examples of each class –three ear conditions and normal case– are shown in Fig 3.

### Features extraction

Feature space representation is performed by filter bank, discrete cosine transform (DCT) and color coherence vector (CCV). Filter bank method has been employed to classify single images using low-dimension features spaces [35]. The DCT can be used to describe textures in the frequency domain from a single image [36]. The CCV has been used as a descriptor that includes information about the color spatial distribution in an image [37]. The filter-bank and DCT methods were chosen due to the capability to generate relevant features in the frequency domain [35, 36]. The CCV method outperforms conventional color descriptors, which are based on compute the color histograms of the image because it includes color spatial information of the image [37]. Following, we explain in detail how each method is used in our work.

#### Filter bank method

**Fig 4:**
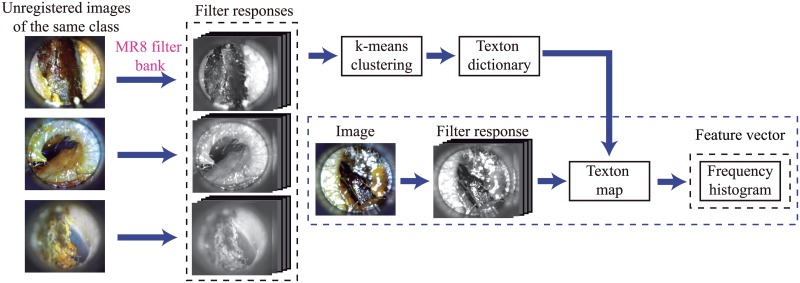
Scheme implemented to generate the feature space based on filter-bank method. The first stage consists in a texton dictionary creation. Such dictionary is used to label the new filter responses to an image from the training set. The second stage computes a histogram of the frequency of each texton occurrence in the labeling stage for each filter response. The frequency histogram is considered as the feature vector.

In this work, we implemented the filter bank presented by [35] to create a texture descriptor, i.e., we used the frequency histogram as features to train a machine learning models. To compute the frequency histogram, we first created a texton dictionary. For this purpose, multiple images from a set of a specific class were convolved with the filter bank. We used a set of eight maximmun filter responses called MR8 filter bank which as proposed by [35]. The resulting filter responses were clustered using the k-means approach [38], and the resulting centroids –called textons– were added to a texton dictionary, which was used for later labeling each filter response to a novel image. Following, we convolved the new image with a filter bank and then labelled each filter response with the texton that lies closest to it, in the filter response space. Finally, we computed a histogram of the frequency of each texton occurrence in the labelling. Further information can be found in [35]. The scheme implemented is illustrated in Fig 4.

We considered 160 images—40 per each class—to build the texton dictionary. To perform the k-means algorithm, we selected *k* = 10 which allows for a low dimensional feature space. The frequency histogram was computed up to 40 bins. Therefore, the dimensionally of the feature space was 40.

#### Discrete cosine transform -(DCT) based texture features

The DCT is similar to the discrete Fourier transform, but uses only real numbers and its performance is comparable to the Karhunen-Loève transform, which is known to be optimum [39]. In applications such as pattern recognition, the DCT has the advantage that most of the signal information tends to be concentrated in a few low-frequency components. We exploit this property to extract the most relevant information –e.g., texture features– of an image.

**Fig 5:**
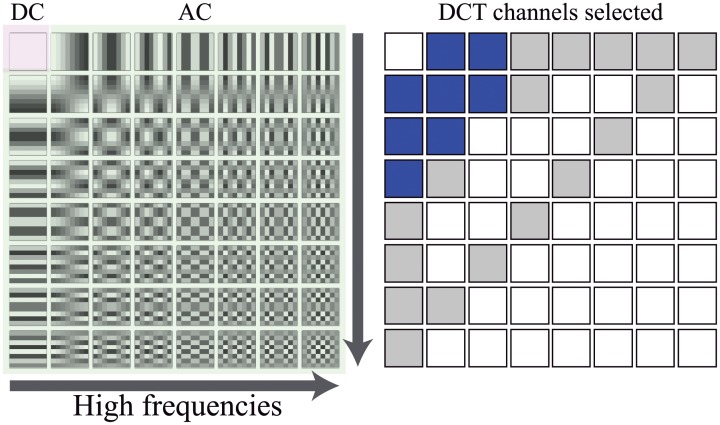
**Left: the 64 basis related to the 8×8 DCT transformation**. The DCT channels that represent the high frequencies are located in the lower right corner. Therefore, they are discarded. Right: Schema used to show the DCT channels selected. Gray background square (including blue ones) represents the DCT channels selected in [36]. Our approach uses only de blue ones.

We adopted the same methodology of the JPEG (Joint Photographic Experts Group) compression algorithm (see [40]) and divided the image into non-overlapping 8×8 pixel blocks. Then, we applied the DCT transform on each block to obtain 64 coefficients. Such coefficients concentrated the image information in the frequency domain. The proportional coefficient to the average pixel values in the 8×8 pixel block is called DC coefficient, whereas the coefficients which describe the variation around the DC value are called AC coefficients as can be seen in Fig 5 (left). Each coefficient represents a channel of the image; therefore, there were 64 channels per image. To use DCT information like features, it needs to be quantized. For this purpose, we design a quantizer assuming that AC coefficients can be modelled statistically as Laplace distribution, as shown in [36]. To make the algorithm more computationally efficient and discard DCT channels that represents the high frequency –likely to be affected by noise– we selected only 8 DCT channels which are illustrated in blue in Fig 5 (right). The selection stage was based on the guidelines provided by [36]. We created a texton dictionary and computed a frequency histogram following the same methodology as the one presented for the filter bank method in the previous section. The frequency histogram based on DCT quantized data is used as features to train the machine learning models.

#### Color coherence vector

Color coherence vector (CCV) can be seen as the degree to which pixels of a certain color are members of large similarly colored regions [37]. The CCV algorithm classifies pixels as either coherent –pixel which is part of a big connected component– or incoherent –pixel which is part of a small connected component. As a result, a color coherence vector represents the pixel classification for each color in the image [37]. To implement the CCV method, the image is filtered to eliminate small variations between neighboring pixels. Then, the color space of the image is quantized such that there are only *N* distinct colors. Finally, each pixel is classified as coherent or incoherent by performing connected components approach [41]. We selected *N* = 27 by performing several tests to achieved a highest validation accuracy in the classification stage. Therefore, the dimensionally of the feature space was 27.

### Machine learning models

In our work, we considered three classification algorithms: support vector machine, k-nearest neighbors and decision trees. Such techniques are considered as supervised learning algorithms that learn from a training set of examples with the associated correct responses [42]. These techniques often provide a high classification performance on reasonably sized datasets [43]. To generate a prediction model, two sets of data were used: the training set to actually train the algorithm and the validation set to keep track of how well it is doing as it learns. After selecting the model with the highest performance, we used a testing set to generate the final results. Following, we explain briefly each algorithm.

#### Support vector machine (SVM)

A support vector machine is one of the most popular algorithms in modern machine learning [42]. Training an SVM model consists of searching the largest radius around a classification boundary (margin) where no data points are placed. The closets points to this margin are called support vectors and are used to define a decision boundary in the classification problem [44]. In a multi-class classification approach, SVM is trained to classify one class from all other –i.e., one-vs-all classification– following the same principle of a binary classification.

#### k-nearest neighbors (k-NN)

The k-nearest neighbor is often selected in classification problems where there is no prior knowledge about the distribution of the data [42]. To make a prediction for a test example, it first computes the distance between such test example and each point in the training set. Then, the algorithm keeps the closest training examples (*k* number of examples) and looks for the label that is most common among these examples.

#### Decision trees (DT)

In decision trees approach classification is carried out by a set of choices about each feature in turn, starting at the root (base) of the tree and processing down to the leaves, where the algorithm delivers the classification decision [23, 42].

### Evaluation metrics

**Fig 6:**
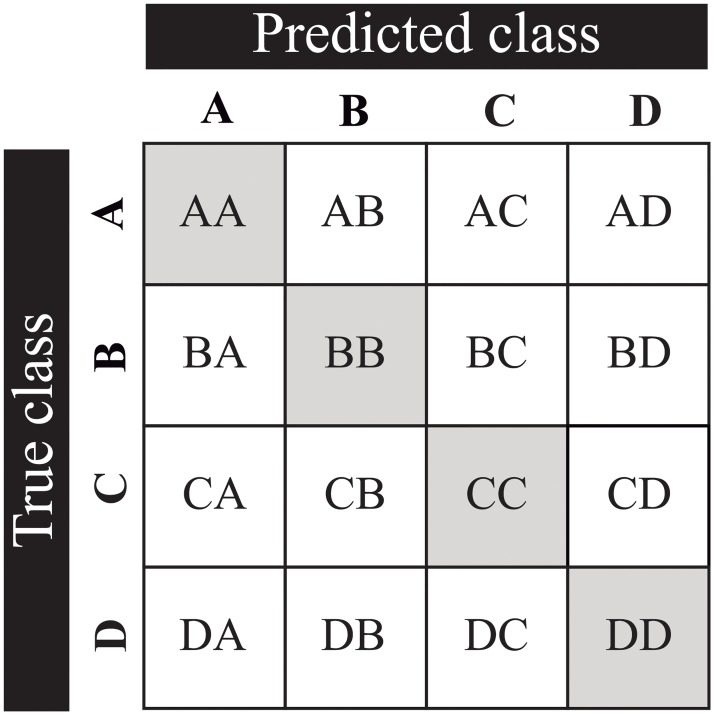
Confusion matrix for multi-class classification problem, with A, B, C and D as classes. The elements located on the diagonal of the matrix represent the correct predicted number of samples; the remaining elements represent the instances incorrectly classified as class A, B, C, or D.

Performance of classification models can be measured from computing a confusion matrix. The confusion matrix indicates the number of instances which corresponds to the predicted and the actual classes. This concept is often used in binary classification [44], but it can be extended to a multi-class prediction where the corresponding class is located on the diagonal of the matrix, whereas the misclassified classes are located outside of the diagonal as can be seen in Fig 6. Briefly, we explain how each metric can be obtained from confusion matrix scores in a multi-class classification approach.

To evaluate the learning models, we computed the accuracy that can be obtained from confusion matrix scores. Furthermore, we also considered other evaluations metrics such as sensitivity, specificity and positive predictive value which are not dependent on the class distributions and therefore will not be biased [44]. The definitions of these metrics in a binary classification approach are extended to multi-class classification using macro-averaging method, as presented in [45].

- **Accuracy** represents the overall effectiveness of a classifier. It can be computed as the average per class as following:
  $$\begin{array}{c} {Accuracy}_{M}=\frac{\sum_{i=1}^{c}\frac{{TP}_{i}+{TN}_{i}}{{TP}_{i}+{FN}_{i}+{FP}_{i}+{TN}_{i}}}{c} \end{array}$$ (1)
- **Sensitivity** (also known as detection rate) quantifies the avoidance of false negatives and demonstrates the capability of a classifier to predict the universe of relevant instances [46]. In a multi-class classification, the sensitivity of the model is calculated as the average among the classes, as follows.
  $$\begin{array}{c} {Sensitivity}_{M}=\frac{\sum_{i=1}^{c}\frac{{TP}_{i}}{{TP}_{i}+{FN}_{i}}}{c} \end{array}$$ (2)
- **Specificity** of a diagnosis model refers to the ability of the test to correctly identify those patients without disease [47]. Specificity of the system is calculated as the average among the classes:
  $$\begin{array}{c} {Specificity}_{M}=\frac{\sum_{i=1}^{c}\frac{{TN}_{i}}{{TN}_{i}+{FP}_{i}}}{c} \end{array}$$ (3)
- **Positive predictive value—PPV** (often called precision) is the ratio between the correctly classified instances from a given class and the total number of instances classified as belonging to that class [46]. The PPV in a multi-class classification approach is averaged among all the classes, as shown below.
  $$\begin{array}{c} {PPV}_{M}=\frac{\sum_{i=1}^{c}\frac{{TP}_{i}}{{TP}_{i}+{FP}_{i}}}{c} \end{array}$$ (4)
  Where *c* represents the number of classes (*i* = 1, …, 4), true positives (TP) means correctly classified class, true negative (TN) means instances correctly classified as not belonging class, false positive (FP) means instances incorrectly classified class and false negative (FN) means all instances that were incorrectly classified as not belonging class.
- **Receiver operating characteristic—ROC** is typically used in binary classification to evaluate the performance of a classifier. It also has been widely used to compare the performance between several classifiers. The horizontal axis represents the false positive rate and the vertical axis contains the true positive rate. We extended the concept for binary classification to multi-class approach through macro-averaging method which consists on drawing a ROC by considering each element of the label indicator matrix as a binary prediction [48]. We use the AUC (Area Under the Curve) value to compare the performance between learning models.

## Results

To obtain a system capable of assisting the physician’s diagnosis, we selected the pair: feature extraction method with learning model that produces the highest validation accuracy, sensitivity, specificity, PPV and AUC.

We generated each learning model using 720 images (180 per class). To perform the classification tests, we randomly splitted the images into a training set (576 images, 80%) and a validation set (144 images, 20%) as can be seen in Table 1. Such random splitting was performed 100 times, following the guidelines previously published in [26, 47]. For each split, we trained the machine learning models using training data set and evaluated each model performance on the validation data set. We summarized the evaluation metrics –i.e., accuracy, sensitivity, specificity and positive predictive value– for each learning model in Table 2. As can be observed that both the SVM and k-NN outperform the decision trees model in terms of accuracy, sensitivity, specificity and PPV, for any of the three feature extraction methods. However, the highest validation accuracy (99.0%) is achieved when the system uses the filter-bank as a feature extraction method and SVM as the learning model. The same pattern is presented in terms of the sensitivity (98.1%), specificity (99.4%), and PPV (98.1%). The k-NN model achieved a close but lower performance than SVM. The validation accuracy was 99.0%, sensitivity was 98.0%, specificity was 99.3% and PPV was 98.1%. The decision trees achieved a lower performance with validation accuracy of 93.5%, sensitivity of 86.9%, specificity of 95.6% and PPV of 87.3%.

**Table 1:** Data distribution for classification test.

Class	Training data	Validation data	Testing data	Total
Earwax plug	144	36	40	220
Myringosclerosis	144	36	40	220
Chronic otitis media	144	36	40	220
Normal	144	36	40	220

**Table 2:** Evaluation metrics of three learning models from classification tests. The highest performance values are achieved using filter-bank method.

	SVM			k-NN			Decision trees		
	Filter-bank	CCV	DCT	Filter-bank	CCV	DCT	Filter-bank	CCV	DCT
Accuracy [%]	**99.03**	96.65	94.10	99.01	97.37	95.49	93.45	91.40	83.86
Sensitivity [%]	**98.06**	93.30	88.19	98.01	94.74	90.98	86.91	82.81	67.72
Specificity [%]	**99.35**	97.77	96.06	99.31	98.25	96.99	95.64	94.27	89.24
PPV [%]	**98.14**	93.51	89.18	98.09	94.98	89.26	87.26	83.26	66.31

**Fig 7:**
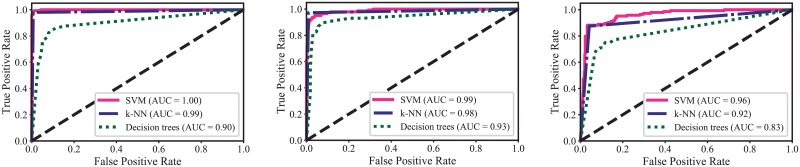
ROC curve and AUC values for three learning models using different feature extraction methods. Left: filter-bank; Middle: color coherence vector; Right: discrete cosine transform.

The ROC curves and AUC values for the three models are shown in Fig 7. Both models, SVM and k-NN, achieved the highest AUC values (1.00 and 0.99 respectively) when the filter bank was used as feature extraction method. However, decision trees model achieved the highest AUC value (0.93) when using features extracted from color coherence vector method.

**Fig 8:**
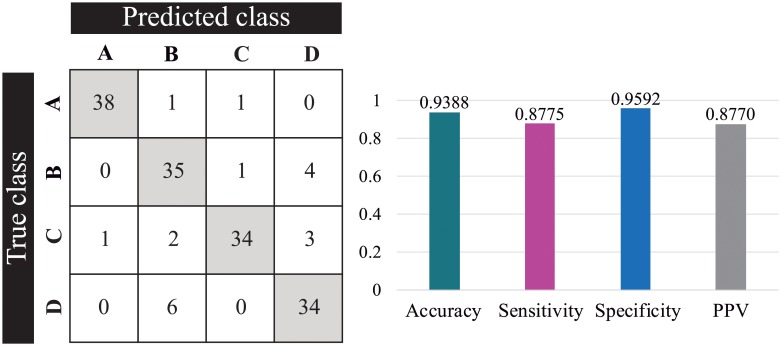
Classification results of SVM model using the testing set after 100 repetitions of the algorithm. Left: confusion matrix for classes A (earwax plug), B (myringosclerosis), C (chronic otitis media) and D (normal); Right: average evaluation metrics.

After comparing the performance of each model, we selected the SVM as a prediction model to assist the physician’s diagnosis in ear conditions such as myringosclerosis, earwax plug and chronic otitis media. Such model was trained using the textures features extracted from filter-bank method. Finally, we performed a classification stage using the testing data set and recorded the statistics as shown in Fig 8. The average accuracy achieved by the proposed system was 93.9%, average sensitivity was 87.8%, average specificity was 95.9% and average PPV was 87.7%.

**Fig 9:**
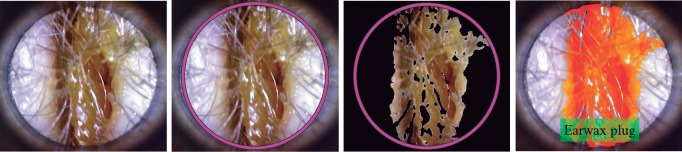
Earwax plug case: If the system classified the RGB input image as earwax plug condition, it highlights the area with wax found in the eardrum or the ear canal. From left to right: RGB input image, region of interest, wax segmentation and area under medical suspicion highlighted.

**Fig 10:**
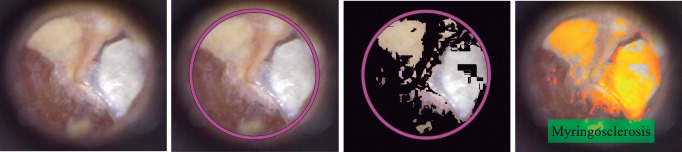
Myringosclerosis case: If the system classified the RGB input image as myringosclerosis condition, the system highlights the white areas found in the tympanic membrane. From left to right: RGB input image, region of interest, myringosclerosis segmentation and area under medical suspicion highlighted.

**Fig 11:**
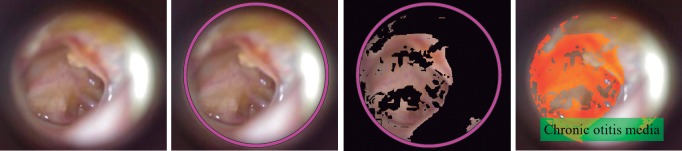
Chronic otitis media case: If the system classified the RGB input image as chronic otitis media disease, the system highlights the perforated area denoted by red color. From left to right: RGB input image, region of interest, chronic otitis media segmentation and area under medical suspicion highlighted.

Furthermore, the proposed system is capable to highlight the area under medical suspicion if the result of the prediction model was any of three ear conditions: earwax plug as can see on Fig 9, myringosclerosis showed in Fig 10 and chronic otitis media illustrated in Fig 11.

## Discussion

The most widely used method by general physicians for making a correct diagnosis of external and middle ear diseases is the visual examination of the eardrum using a manual otoscope. However, in many occasions, general practitioners do not have enough training and experience for making correct diagnosis. In addition, due to economic or geographical limitations, not all population can have access to an ENT (ears, nose and throat) specialist for an adequate diagnosis –for example, in Chile, there is a significant gap between the population and the number of specialists available [49]. Therefore, auxiliary tools are needed to help general practitioners to improve diagnostic accuracy and reduce their number of referred cases.

We developed a computer-aided support system to assist the physician’s diagnosis, in particular, to distinguish between a normal ear and three different frequent ear conditions: myringosclerosis, earwax plug, and chronic otitis media. To ensure a certain level of diagnostic accuracy comparable to that by ENT specialist and provide patients with optimal care, we selected the most suitable feature extraction method and learning model that achieved the highest validation accuracy. The results showed that the highest performance of the learning models was obtained using texture information processed by the filter bank method. However, the features extracted from the other two methods were suitable to use in the training stage. In the classification tests using validation data, SVM and k-NN models showed the highest performance over decision trees model. The validation accuracy of SVM model was 99.0%, sensitivity was 98.1%, specificity was 99.4%, positive predictive value was 98.1% and AUC was 1.00. Moreover, the classification tests using testing data showed that the proposed system achieved an average accuracy of 93.8%, average sensitivity of 87.8%, average specificity of 95.9% and average positive predictive value of 87.7%. Classical machine learning techniques such as SVM, k-NN and decision trees have provided high performance in classification tasks, in particular in reasonably sized datasets [43]. These techniques are easy to interpret, which simplifies the adjustment and calibration of the model. There are other more complex models, such as convolutional neural networks, that can be used to address the same challenges, but the size of the database must be increased to achieve comparable performance. These models will be explored in future work.

Previous works in automatic otoscopic diagnosis have failed to achieve more than 90% of accuracy. We can mention [23, 24] which used a binary classification approach of still color images of the eardrum to classify between normal and otitis media cases. They achieved an accuracy of 73.1% and 68.3% respectively. Both cases exploit color information to train learning models. However, they concluded that color alone does not provide a sufficient discriminative for the classification problem. Another related study that classified normal ear and otitis media cases was proposed in [25]. Such a study used color, texture, and geometric information to train a support vector machine and improved the accuracy (88.1%) achieved by the previous authors. However, the specificity, ability of the system to correctly reject healthy patients without a condition was 79.9%. A more recent study [28] implemented a system to diagnose the tympanic membrane as normal, otitis media cases or ear canal obstructed. The accuracy achieved was 86.8%. We notice that the previous studies do not clarify if the results were achieved by performing a classification stage using the validation or testing sets.

A more complete study to classify middle ear diseases was presented in [26]. Although the results were presented as a binary classification like a normal and abnormal case, the methodology included specific information for each disease such as tympanosclerosis, perforation, cerumen, retraction and post-injection crust. It achieved accuracy and specificity near 84.6%.

We also mention studies based on deep learning approaches which achieved a higher accuracy. An approach to report the condition of the eardrum as normal or abnormal was presented in [50]. Such study used two different deep learning architectures and achieved an accuracy of 84.4%, sensitivity of 85.9%, and specificity of 82.8%. Other study [51] proposed a diagnosis system based on deep convolutional neural networks that achieved an average accuracy of 93.6%. Such study classified five categories of ear diseases: attic retraction, tympanic perforation, myringitis, otitis externa and tumor, and included normal cases. However, the results showed that the performance of the model –i.e., accuracy– depends on the number of the images for training. The performance is reduced if the number of images decreases (10000/93.6%, 5000/86% and 2000/80%). This is a common limitation of deep learning approaches; the model is biased by database. Furthermore, not always a very large databases are available, especially in the field of otolaryngology where the few databases that exist are restricted.

We compare our results with previous works mentioned in terms of accuracy, sensitivity, specificity, and positive predicted value (PPV) as shown in Table 3. We can observe that the classification accuracy achieved outperforms what is reported in the literature. Only sensitivity value of [25] is higher than the proposed model, but such work does not specify if the results were achieved using testing or validation sets. Furthermore, the accuracy and the specificity of [25] are lower than the proposed model.

**Table 3:** Comparison of previous works and the proposed model.

	Minorica [23]	Vertan [24]	Shei [25]	Senaras [26]	Myburgh [28]	Senaras [50]	Cha [51]	Ours
Accuracy	73.1	68.3	88.1	84.6	86.8	84.4	93.6	**93.9**
Sensitivity	-	-	91.6	87.3	87.0	85.9	-	**87.8**
Specificity	-	-	79.9	81.4	96.4	82.8	-	**95.9**
PPV	-	-	-	-	87.4	-	-	**87.7**
Classes	2	2	4	2	5	2	6	**4**

In order to create the ear imagery database, we performed a real-life otoscopic video acquisition, in similar conditions of a normal otoscopy during clinical practice. We had manual focus (not autofocus) and no stabilization, thus we experienced some blurred videos and a few frames per video on focus. Although, it might be considered a limitation of our work, the continuous acquisition through video-otoscopes allowed us the design of assisted diagnosis system while something very similar to regular otoscopy is performed. Additionally, we agree that the quality of images would be better with other devices such as oto-endoscopes, however, otoscopes are cheaper and used worldwide. The video otoscope that we used was already approved by the FDA. Therefore, our proposed system, which includes an assisted video otoscopy, would have an easier implementation and a major impact on primary health care. The blurred images can also be analyzed and treated by AI algorithms, thus, not having the whole video images on focus it is not necessarily a problem for diagnosis or classification accuracy, even though we did not assess it directly. Although a large sample of cases was randomly selected, we still do not have the full diversity of chronic otitis media cases, such as cholesteatomas. Therefore, there is a potential bias in the selection of the images. However, to avoid it and to bring our data closer to reality, our database included images with different projections of eardrums and ear canal, where the skin or eardrum color varied. Furthermore, the images were taken in three different attention boxes –each one equipped with a digital otoscope and a computer– thus diversifying the data.

Finally, we evaluated only four conditions present at otoscopy, and clinically there are several other relevant conditions to include in diagnosis. It is not clear if our approach would decrease in accuracy if more conditions are included. Thus, a more detailed evaluation of otoscopic features should be performed in future studies. Nevertheless, compared to the literature, our study gets a higher accuracy by having conditions that have not been evaluated before such as myringosclerosis and earwax plug, in real clinical environments.

In our future studies, we will integrate other ear pathologies that are less frequent, but their diagnosis can represent a challenging even for a specialist. Additionally, we will train other learning models in particular deep convolutional neural networks that might allow integrating more class maintaining high performance. Artificial intelligence is increasingly having an impact on the practice of medicine. The specialists are key actors in the clinical integration between the currently established methods and based-AI technologies that will improve patient care.

## Conclusion

We developed a computer-aided diagnosis system for four ear conditions: normal, earwax plug, myringosclerosis and chronic otitis media. The system achieved an accuracy of 93.9%, sensitivity of 87.8%, specificity of 95.9% and positive predictive value of 87.7%. Such results outperform what is reported in the literature. The system was capable to highlight the area under medical suspicion if the system response was any of the ear conditions, except in normal case. The system might be used for general practitioner to make more accurate diagnosis using a low-cost system based on machine learning models, vision processing techniques, and a digital otoscope (FDA approved).
